# Image dataset of important grape varieties in the commercial and consumer market

**DOI:** 10.1016/j.dib.2023.108906

**Published:** 2023-01-20

**Authors:** Lafta R. Al-khazraji, Mohammed Abdallazez Mohammed, Dhafar Hamed Abd, Wasiq Khan, Bilal Khan, Abir Jaafar Hussain

**Affiliations:** a Ministry of Education, General Directorate of Education, Salah al-Din, Iraq; b University of Karbala, College of Computer Science and Information Technology, Information Technology Department, Iraq; c Department of Computer Science Al-Maarif University College, Alanbar, Iraq; d Liverpool John Moores University, Liverpool L3 3AF, UK; e School of Computer Science and Engineering, California State University San Bernardino, 5500 University Parkway, San Bernardino, CA 92407, USA; f Department of Electrical Engineering, University of Sharjah, Sharjah, UAE

**Keywords:** Pattern recognition, Classification, Deep learning, Cultivation, Grape's type

## Abstract

This work presents a primary dataset collected from various geographic locations in Iraq for the seedlings of eight varieties of grapes that are used for local consumption and export. Grape types included in the dataset are: deas al-annz, kamali, halawani, thompson seedless, aswud balad, riasi, frinsi, shdah. Leaves of each type of the seasoned fruit were photographed with high resolution device. A total of 8000 images (i.e., 1000 images per category) were captured using random sampling approach while maintaining the balance and diversity within grape image data. The proposed dataset is of significant potential impact and usefulness with features including (but not limited to) 8 varieties, that have different tastes and can support various industry in agriculture and food manufactures.

**Specifications Table**

**Table:** 

Subject	Computer Science
Specific subject area	Computer Vision, Pattern Recognition, machine learning, deep learning
Type of data	Images
How the data were acquired	Grape seedlings were captured via a digital camera device with the following specification: Nikon 7200 Crib Frame 24 MP DX CMOS sensor. Expeed processor 4. 3.2-inch tilting LCD display. MultiCAM 3500DX2 focus system with 51 points. Lens used 35 mm f 1.8.
Data format	Raw
Description of data collection	Grape seedlings were captured in different regions of Iraq for eight common types that are desirable in the local market and for export. Useful information of the parts of the photographed seedling (i.e., high resolution images of leaves) is provided in the dataset that is typically used to identify grapefruit types. Seedlings were photographed during the fruiting season to identify their types with highest confidence. Dataset for 8 included classes (deas al-annz, kamali, halawani, thompson seedless, aswud balad, riasi, frinsi, shdah) comprises of a total of 8000 image, with 1000 images for each grape type.
Data source location	Country: Iraq, Governorate: Salahaddin, cites: al_dujail , balad , Ishaqi, Yathrib . Between these two coordinates 33° 30′ 34″ N 044° 14′ 02″ E 34° 00′ 58″ N 044° 08′ 44″ E
Data accessibility	The proposed dataset is publicly available at Mendeley platform as described below: Repository name: Mendeley Data identification number: Direct URL to data: https://data.mendeley.com/datasets/7n3d6696hz/2

## Value of the data

• This dataset can be proven useful for the identification of the types of grape seedlings mainly before their plantation and growth [1,2].
• The early identification of the desired grape type is critical due to the long seeding duration (approximately # of years) along with the effort and cost associated with their growth [3].
• Conventional methods for the identification of the type for a seedling requires expert knowledge while at the seeding site where the effort and cost associated with the involved labor can be significant [4].
• The development of a classification model for grape class identification with higher accuracy can be an important undertaking providing significant advantage with respect to the economic factors as well as timely identification.
• The proposed dataset can be proven useful for computer science community, particularly computer vision, machine learning and deep learning to build robust grape classification models that could accurately classify grapes of various types.
• Models of such nature can be utilized by farming community before plantation to cut the time and cost associated with planting grapes of the desired types and reduce the risks of planting incorrect types. Additionally, the proposed dataset provides an opportunity to the research community to build machine or deep learning-based classification models for the detection of the plant health status (i.e., diseases free plants) for the included types of grapes.

## 1 Objective

To reduce the time, cost, and effort in identifying the desired seedlings of grape cultivars prior to planting, the proposed dataset is configured to build a model capable of classifying eight cultivars of grapes desired in Iraq.

## 2 Data Description

**Fig. 1:**
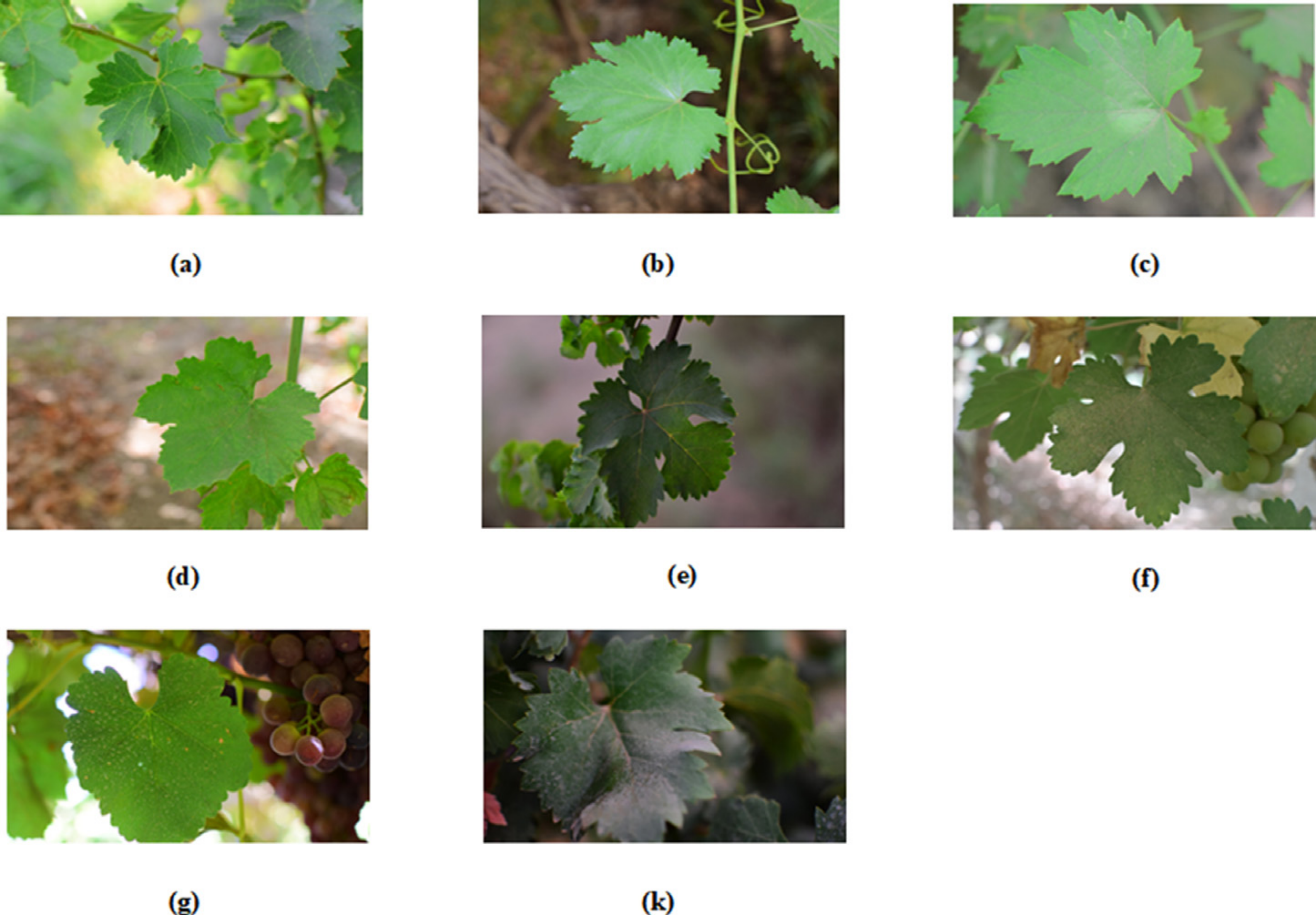
*Examples of each of the included grape class as follows:* (a) deas al-annz's leaf class, (b) *kamali*’s leaf class, (c) *halawani*’s leaf class, (d) thompson seedless's leaf class, (e) *aswud balad* ‘s leaf class, (f) *riasi* ‘s leaf class, (g) *frinsi* ‘s leaf class and (k) *shdah* ‘s leaf class.

The dataset consists of eight folders (corresponding to the included grapes types), each folder contains 1000 images of JPG format. The images within the dataset are all of the resolution of 6000 × 4000 pixels. Because of the high resolution of the images, the size of the images became 49.8 GB, which is a large size for uploading and downloading the dataset from the Internet, so the resolution of the images was changed using a tool resize pictures in Windows to be the dimensions 1620 × 1080 pixels, Thus, the data size is 1.95 GB, After compressing with a zip program, the data size became 1.83 GB.Each folder represents one of the eight grape varieties (Deas Al-Annz, Kamali, Halawani, Thompson Seedless, aswud balad , riasi ,frinsi, shdah). The grape leaves have been photographed as shown in Fig. 1.

## 3 Experimental Design, Materials and Methods

**Fig. 2:**
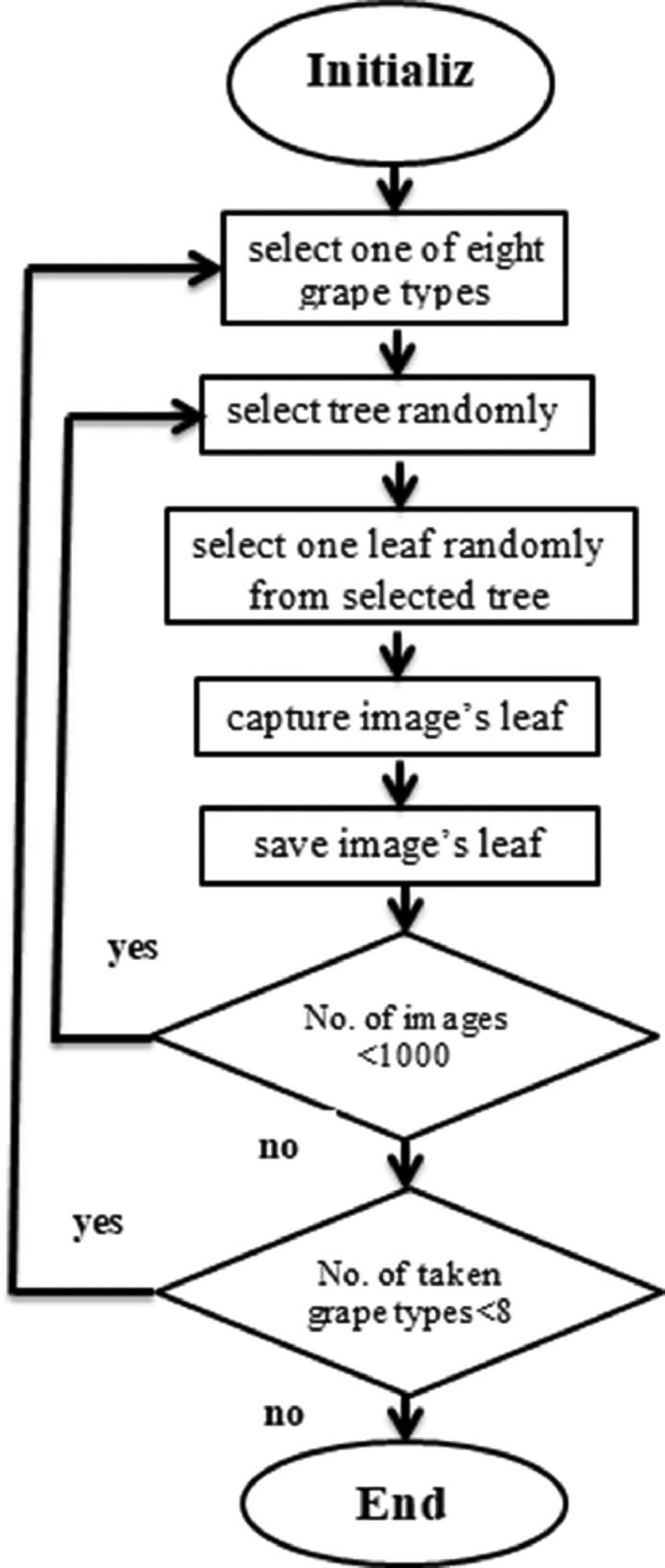
Workflow for the acquisition of the images of grapes of each type based on the random selection of the tree for each grape class.

Acquisition of the images for each grape type followed the workflow of based on a random leaf selection approach as illustrated in Fig. 2. In the proposed approach, selection of the constituent of the population of each grape type followed the uniform distribution with an equal probability of being selected.

Data diversity was maintained via random leaf selection approach, by which both the leaves to be photographed and tree for each grape type were selected randomly.

In the post image acquisition process for each grape type, images per each grape type were transferred from the camera's memory to an external hard drive and kept in a folder bearing the name of the grape type. The acquisition of the images for the next grape type would then follow after the removal of the images for the transferred grape type, and so on. Leaves containing dust were not photographed at a high rate, as they represent noise, and the leaves that were eaten were not photographed. Photographed with the knowledge and consent of the owners of the gardens.

## Ethics Statement

It should be noted that the work meets the ethical requirements.

## CRediT authorship contribution statement

**Lafta R. Al-khazraji:** Conceptualization, Methodology, Software. **Mohammed Abdallazez Mohammed:** Data curation, Writing – original draft. **Dhafar Hamed Abd:** Visualization, Investigation, Supervision. **Wasiq Khan:** Writing – review & editing, Conceptualization. **Bilal Khan:** Writing – review & editing, Data curation. **Abir Jaafar Hussain:** Writing – review & editing, Methodology.

## Declaration of Competing Interest

The authors declare that they have no known competing financial interests or personal relationships that could have appeared to influence the work reported in this paper.

The authors declare the following financial interests/personal relationships which may be considered as potential competing interests.

## Data Availability

Grape Varieties Dataset (Original data) (Mendeley Data).

## References

[1] Yang Z., Tian J., Wang Z., Feng K. (2022). Monitoring the photosynthetic performance of grape leaves using a hyperspectral-based machine learning model. Eur. J. Agron..

[2] Gao F., Hao X., Zeng G., Guan L., Wu H., Zhang L., Wei R., Wang H., Li H. (2022). Identification of the geographical origin of Ecolly (Vitis vinifera L.) grapes and wines from different Chinese regions by ICP-MS coupled with chemometrics. J. Food Compos. Anal..

[3] Malwe P.D., Gawali B., Deshpande M., Panchal H., Darade P. (2022). Energy nexus for grapes production: a case study of Sangli region in India. Energy Nexus.

[4] Charlton D., Rutledge Z., Taylor J.E., Christopher B., Barrett David R. Just (2021). Handbook of Agricultural Economics.

